# Sustaining a pharmacist-led COPD transition of care service in the rural context: a qualitative review of multilevel stakeholder perspectives from two Veterans Affairs medical centers

**DOI:** 10.3389/frhs.2026.1715917

**Published:** 2026-02-05

**Authors:** Edward C. Portillo, Tiffany Parham, Martha Maurer, Dylan Erdelt, Jenna Vande Hey, Nora Jacobson, Steven Do, Jennifer Nguyen, Sarah Will, Heather Ourth, M. Shawn McFarland, Michelle A. Chui

**Affiliations:** 1 School of Pharmacy, University of Wisconsin-Madison, Madison, WI, United States; 2 William S. Middleton Veterans Affairs Hospital, Madison, WI, United States; 3 Institute for Clinical and Translational Research, School of Nursing, University of Wisconsin-Madison, Madison, WI, United States; 4 Clinical Pharmacy Practice Office, Department of Veterans Affairs Clinical Pharmacy Practice Office, Washington, DC, United States; 5 School of Nursing, University of Wisconsin-Madison, Madison, WI, United States

**Keywords:** care transitions, clinical pharmacist, COPD, implementation science, interprofessional care teams, rural, sustainment, Veteran health

## Abstract

**Introduction:**

Chronic Obstructive Pulmonary Disease (COPD) is a leading cause of morbidity and mortality globally, and rural patients are at higher risk for poor COPD health outcomes. The United States National Institutes of Health (NIH) has issued a call for innovative care delivery models to address this gap in COPD care quality in rural communities. This paper explores the perspective of healthcare teams at two rural VA medical centers sustaining an innovative interprofessional COPD care delivery model, COPD Coordinated Access to Reduce Exacerbations (CARE).

**Methods:**

This qualitative evaluation was conducted at two rural VA medical centers in the Southeast and Midwest regions of the US. Eleven semi-structured interviews with diverse clinical stakeholders were conducted including: facility leaders, hired pharmacists, clinical pharmacists, nurse care managers, primary care providers, and referral champions. The Practical Robust Implementation and Sustainability Model (PRISM) was applied to develop interview guides and informed data analysis. Interviews were transcribed and a mixed inductive-deductive approach was used for data analysis, involving iterative coding and consensus-building among five evaluators to identify emerging themes.

**Results:**

Five overarching themes emerged as key facilitators of COPD CARE sustainment including (1) leadership support, (2) value alignment, (3) service institutionalization, (4) service adaptation, and (5) interprofessional collaboration. Notably, clinical pharmacists were described as filling care gaps in these under-resourced facilities.

**Conclusion:**

This evaluation demonstrated the impact of clinical pharmacists serving as prescribers in improving COPD management in rural settings. The sustainment factors identified can be utilized to inform the expansion of similar, team-based healthcare programs across rural settings.

## 1 Introduction

Chronic Obstructive Pulmonary Disease (COPD), defined by persistent, irreversible respiratory symptoms, is a leading cause of morbidity and mortality globally. Flares of COPD, termed COPD exacerbations, are common and associated with 5-year mortality rates above 50% (1). Rural patients are among the most likely to have a COPD diagnosis and to experience negative COPD outcomes (2, 3). The United States (US) National Institutes of Health (NIH) recognizes this gap for rural patients with COPD and has issued a call for innovative care delivery models (4, 5).

This paper explores the perspective of healthcare teams engaged in providing an innovative interprofessional COPD care delivery model called COPD Coordinated Access to Reduce Exacerbations (CARE). COPD CARE integrates clinical pharmacists as prescribers to promote the delivery of COPD evidence-based practices. To the authors’ knowledge, it is one of the largest programs that integrates pharmacists within primary care teams to manage COPD. Using a national implementation package termed “The Academy” that includes training resources, informatics tools, and additional program infrastructure designed using principles from the field of Dissemination and Implementation (D&I) science, the program has been scaled to over 50 Medical Centers in the Department of Veterans Affairs, the largest integrated healthcare system in the US. Since 2020, it has served over five thousand patients following a COPD exacerbation. COPD CARE is funded by the VA Office of Rural Health (ORH) to expand across its network of rural medical centers.

While the implementation of COPD CARE has been studied, and barriers to implementation in rural medical centers have been identified (6–11), the sustainment of COPD CARE at rural medical centers has not yet been explored. The need to better understand how to sustain evidence-based practices is clear, with sustainment being described as “one of the most significant translational research problems of our time” (12). Only 23% of health programs are sustained within 24 months following implementation, resulting in wasted healthcare spending and clinician time, as well as missed opportunities to provide high-quality care to patients (13).

Many stakeholder groups are needed to deliver COPD CARE effectively because the program requires coordination across multiple clinical service lines and leadership support. Insights from stakeholders are critical to better understand factors that promote service sustainment. This assessment sought to learn from stakeholders at high-performing rural VA medical centers what factors facilitated sustainment of their COPD CARE practice. Findings will inform sustainment efforts for COPD CARE practice nationally.

## 2 Methods and materials

### 2.1 Site selection

This evaluation was conducted at two VA medical centers located in the rural Southeast and Midwest regions of the US. Each medical center was a top performer, relative to other VAMCs in the cohort, in sustaining the COPD CARE program, with high rates of patient visits, timely care delivery, and fidelity to quality metrics observed using a program performance dashboard.

### 2.2 Interview guide development

Six semi-structured interview guides were developed to engage with stakeholders playing different roles in COPD CARE. Interview guide design was informed by the Practical, Robust, Implementation and Sustainability Model (PRISM), a framework designed to guide and evaluate the implementation of evidence-based interventions (14). The PRISM framework was selected due to its emphasis on stakeholders with unique vantage points in programmatic delivery. This evaluation focuses on one dimension of the PRISM framework, termed organizational perspective.

### 2.3 Study design and participants

**Figure 1:**
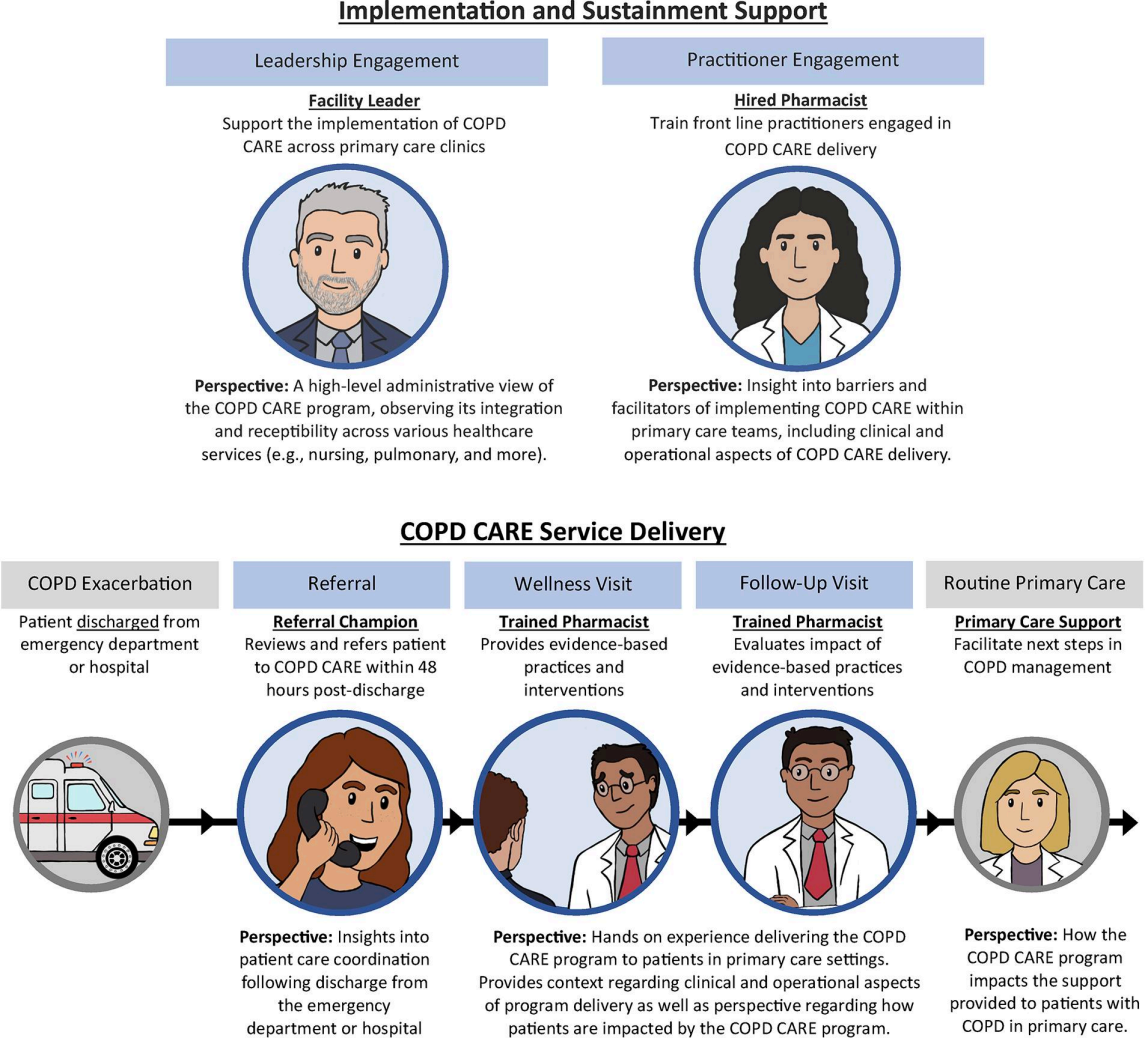
Illustrates the perspective of interprofessional team members on the implementation, sustainment, and delivery of COPD CARE. Implementation and sustainment support for the COPD CARE program are provided by Facility Leaders and Hired Pharmacists who bring high-level administrative and clinical perspective. A Referral Champion provides insight on care coordination and a Trained Pharmacist provides perspective on clinical care delivery. Additional team members within the routine primary care team describe how the COPD CARE program impacts support provided to patients.

Medical center leadership and clinicians, including facility leaders, hired pharmacists who served as clinical leaders, trained pharmacists, nurse care managers, primary care providers (PCPs), and referral champions, were invited to participate in interviews. Hired pharmacists train front-line practitioners in COPD CARE delivery and provide high-level administrative and clinical perspective on implementation facilitators and barriers. Trained pharmacists deliver COPD evidence-based practices to patients and offer hands-on experience delivering the COPD CARE program. The roles of additional participants and descriptions of each role are detailed in Figure 1. Participation in interviews was voluntary and coordinated in collaboration with pharmacists hired at each medical center to implement the COPD CARE program. A participant may have held multiple stakeholder roles in their respective medical centers. Interviews were conducted in Spring of 2024, 18 months following COPD CARE implementation, by a member of the evaluation team (MM) virtually using Microsoft Teams^TM^. A total of 11 interviews with stakeholders were conducted. A total of two facility leaders, two trained pharmacists, two nurse care managers, two primary care providers, and three hired pharmacists, two of whom also served as referral champions, participated in interviews.

This evaluation was conducted to inform COPD CARE delivery across the Department of Veterans’ Affairs and was deemed quality improvement using the University of Wisconsin-Madison IRB's “QI/Program Evaluation Self Certification Tool.”

### 2.4 Analysis

Each interview was audio recorded, de-identified, and transcribed by a professional transcription services program at the Salt Lake City VA Medical Center. Six evaluators (MM, EP, TP, DE, JVH, and NJ) with collective experience in qualitative analysis, clinical management of COPD, and program implementation comprised the evaluation team. A mixed inductive-deductive approach to analysis was completed through iterative, independent coding and consensus building to identify emerging themes. The role of each stakeholder participant, medical center location, and corresponding PRISM domain were tracked throughout the coding process. Coding discrepancies among evaluators were discussed until a consensus was reached. To confirm saturation, a rolling analysis of new codes was maintained throughout the data collection period. Evaluators used Microsoft Excel™ to facilitate the organization of codes and identification of themes. Group discussions about how, why, and when codes were applied were documented through audit trails. As higher-order themes emerged, data displays and memoing were iteratively developed and shared among the research team. In this way, the themes were validated and refined.

## 3 Results

Five overarching themes were identified as sustainment facilitators for COPD CARE: (1) value alignment, (2) leadership support, (3) service institutionalization, (4) service adaptation, and (5) interprofessional collaboration.

### 3.1 Value alignment

Strong alignment between COPD CARE and the VA's values of commitment, advocacy, and excellence was a sustainment facilitator. Participants described COPD CARE as reinforcing the following organizational priorities:

#### 3.1.1 Population management

COPD CARE strengthened the VA's population management efforts by utilizing strategies to identify and treat patients at high risk of poor disease outcomes. Quality of care was evaluated using Healthcare Effectiveness Data and Information Set (HEDIS), a set of standardized performance measures.

“[The clinical pharmacist] just kinda gets in and sees who are our worst patients and it's really helped our, our Veterans get better control in their chronic diseases. And it’s helped our numbers as well, the HEDIS number’s different, monitors they’re looking at. But she’s, helps keep people out of the hospital.” [Primary Care Provider]

#### 3.1.2 Effective medication use

Over 70% of patients with COPD in the US do not use their inhalers as prescribed, highlighting the need for inhaler technique education (15). COPD CARE was frequently described as promoting effective patient inhaler use and overall medication management.

“I can't tell you how many [patients] we had that had no idea how to use their inhalers or didn't know what was in them. And to see the way that, that she [clinical pharmacist] can pull that information out of them … to get to the problem because they didn't know that the problem with the medication. You know, they thought that they were using it correctly the whole time. So for her to be able to talk with them and show them exactly what it is and how to use it has made a huge difference in their lives for sure.” [Nurse Care Manager]

“…We kind of assume you know how to use [inhalers], or you’re using them correctly. That’s probably the biggest benefit I can think of is just having that contact… Maybe that lack of understanding played a role in the exacerbation itself, and maybe with that education, something like that, it could, you know, future exacerbations could be avoided or prevented.” [Trained Pharmacist]

#### 3.1.3 Care transitions

Patient care transitions following hospital discharge were described as having been improved with COPD CARE delivery through enhanced follow-up and continuity of care that is often lacking in standard post-discharge processes.

“Again, with some of these follow-ups, it’s like if we [pharmacists] weren’t doing it, I don’t know if anybody else would be checking in on these inhalers. I think for a lot of patients, maybe that’s all that’s needed is just that little extra follow-up” [Trained Pharmacist]

“They go to a doctor and they get told they have this [disease] and they get released from the hospital and that’s, that’s just it. So knowing that they need that follow-up care, even if they see a pulmonologist, that they may not be getting.” [Nurse Care Manager]

### 3.2 Leadership support

Clinical pharmacists, PCPs, and nurses all described the benefits of a dedicated lead pharmacist. This leadership role, supported by funding, effectively trained team members and fostered interprofessional service lines.

The VA Office of Rural Health provided funding that supported hiring at least one lead pharmacist at each rural medical Center to serve as a resource to successfully scale COPD CARE across all facility primary care teams.

“So she [Clinical Pharmacist Leader_Hired Pharmacist] was really so key to making me feel comfortable in being able to address that disease state ‘cause I hadn’t done that in my career. And so she gave us really good education and she showed us the resources and she sat in our first visits, our first couple visits with us.” [Trained Pharmacist]

“She’s [Clinical Pharmacist Leader_Hired Pharmacist_Referral Champion] given us the little updated handouts on what the appropriate inhalers are to use … she presented an education book and so that’s been helpful. And so she’s just trying to keep us up to speed with the guidelines and different treatments. So she’s just been a real help.” [Primary Care Provider]

“I’d give ‘em the resources, obviously, you know, just to brush up on COPD disease state ‘cause this is definitely a new one for many of them…I always said that I have an open door. Just reach out for any questions. If you need guidance for any help in inhaler selection or anything, definitely to ask me, and a lot of ‘em do.” [Hired Pharmacist and Referral Champion]

Leadership support across interprofessional service lines was described as a factor that “*has contributed to our success,”* by allowing different departments to work together to integrate COPD CARE into existing workflows and address operational challenges.

“[Our] chief of respiratory has been great…he’s been helpful with nebulizer education, and I’ve worked with prosthetics to see how the nebulizers are being mailed and delivered and education for those…he has been helpful with resources and sending me things and making decisions.” [Clinical Pharmacist Leader_Hired Pharmacist and Referral Champion]

“…You have to continue to work to get those providers on board because you can have very enthusiastic pharmacists or nurses or something like that with the program, but if you don't get your provider on board, in the long run, it’ll die out after a while… [Early adopters] get the support from the leadership team, but it’s selling it to that middle cohort beyond your early adopters that you really need to continue to maintain the program and keep it goin’.” [Facility Leader]

### 3.3 Service institutionalization

High staff turnover in rural settings is detrimental to patient care delivery and negatively impacts the sustainment of healthcare programs. To combat this, COPD CARE training and resources for care delivery were embedded within the VA's new employee onboarding and continued education. This approach, providing proper training and establishing work culture, contributed to sustainment.

Clinicians reported integrating COPD CARE training was effective in mitigating staff turnover while also promoting continuous program awareness and utilization.

“And plus, our primary care provider turnover, so in a way, we kind of have to make sure to continue to deliver the education to all the primary care teams to let them know, hey, this program is this. Please utilize us, and our pharmacists all can deliver COPD care.” [Clinical Pharmacist Leader]

“So just making sure all of the new providers that are coming on board are aware of the program [COPD CARE]. Marketing. Speaking at [primary care] meetings and med staff meetings as updates were being made.” [Clinical Pharmacist Leader, Hired Pharmacist, and Referral Champion]

Celebrating program successes and achievements demonstrated the positive impact of COPD CARE to build morale and cultivate a supportive work environment.

“And so you’ve gotta kinda create an environment where you leverage those early successes that you have and that you’re able to come, and you say, hey, look. Here’s the data that supports us doin’ what we’re doin’ to convert that. So it’s kinda that, you know, you got the people in the trenches that are committed. They see it. They’re committed to instituting it. They get the support from the leadership team, but it’s selling it to that middle cohort beyond your early adopters that you really need to continue to maintain the program [COPD CARE] and keep it goin.” [Facility Leader]

### 3.4 Service adaptation

COPD CARE's sustainment was facilitated by its adaptability, both in utilizing existing VA services and in tailoring the program to overcome rural infrastructure limitations. There were several examples of adaptations made to enhance COPD CARE delivery at local medical centers.

Many adaptations were unique to the rural context where limited, or even absent, pulmonary services necessitated collaboration with other, larger VA medical centers.

“Most of our patients—we don't have specialized care here. We don't have a pulmonologist, so even if we are getting PFTs done here, they’re being read by a pulmonologist out of the [southern city] VA..” [Clinical Pharmacist Leader, Hired Pharmacist, and Referral Champion]

“So if we needed a pulmonologist, they're gonna have to be seen in the community [outside of VA]. Just because the, we just don't have those assets to our, to our facilities. A lot of other VA clinics are, are a lot bigger, so they have that.” [Nurse Care Manager]

New collaborations with Community Care Coordinators, liaisons between the VA and non-VA care, were also formed to help identify and refer patients. These relationships were important to identifying patients who needed to receive emergency care outside of the VA.

“So I had to work with the community care department to coordinate a notification system, which has worked well, but it doesn’t capture every single patient. So what we’re doing currently is the community care nursing staff is tagging me on any exacerbation, COPD exacerbation, and then I do the referral review just like I would do for any patient that showed up on the dashboard. [Clinical Pharmacist Leader, Hired Pharmacist, and Referral Champion]

### 3.5 Interprofessional collaboration

Provider shortages and high workload demands are pervasive in rural healthcare settings. Stakeholders described the COPD CARE delivery model as addressing many workforce deficits through interprofessional collaboration, leveraging the diverse skills of different team members.

COPD CARE's collaborative nature addressed critical staffing limitations, preventing fragmented care and ensuring consistent follow-ups for patients with COPD.

“Our pulmonary provider retired last year, so facilitating primary care setting, not many people really, especially the [primary care providers] they are already busy. They just don't really have time to go out into COPD care. So in a way, I think this program worked for us because our [primary care team] pharmacists… can identify the stable patient with established diagnosis and manage their COPD medication regimen” [Clinical Pharmacist Leader]

“I’m definitely addressing patients who would, I would consider a loss to follow up, that have been lost to follow up for years, which has led to that exacerbation, I think, in my opinion. They've been lost, they got hospitalized, and now they're being brought back into care and getting everything addressed and being seen again. And they were lost to care because there are not providers in this clinic.” [Trained Pharmacist]

Pharmacist management of COPD was a change in the primary care delivery model for these medical centers, as clinical pharmacists were not as involved in COPD management prior to the implementation of COPD CARE. Stakeholders described the integration of a clinical pharmacist to provide COPD disease state management as “*a tremendous asset*” that improved patient care.

“I think with the amount of knowledge that [the Clinical Pharmacist Leader] has… and that she shared with us as the nurses, that helps us in turn share that information with our Veterans in a way that they can understand what’s going on. Which has, which has also decreased the amount of hospitalizations for exacerbation because now they know their disease process, they know what’s causin’ them to be short of breath, they know what to do with their medications, how to take them, when to take them.” [Nurse Care Manager]

“…Since I work with a lot of Veterans with dementia, she’s [Hired Pharmacist] helped a lot with switching ‘em to the nebs, and sometimes it’s hard because I need special approval like to be put on budesonide. And it just helps when she’s seen ‘em to get the nonformularies approved…and so it’s helped that process of getting the right med ordered and approved and educated the wife.” [Primary Care Provider]

## 4 Discussion

The sustainment facilitators identified by stakeholders at two rural VA medical centers in different regions of the US were consistent with evidence provided by D&I science, including the importance of organizational characteristics (e.g., supportive leadership, engaged stakeholders) (16–18), intervention characteristics (e.g., impact, adaptability) (18, 19), and implementation processes (e.g., ongoing training, local adaptation) (18, 20, 21). These practical, real-world examples of facilitators to program sustainment can inform replication and scale-up efforts in similar rural medical centers across the US.

Successful sustainment of COPD CARE was partly due to its strong congruency with core VA values: commitment, advocacy, and excellence. COPD CARE reinforces commitment and excellence by demonstrably improving patient outcomes and thus strengthening the commitment of healthcare professionals to further advocate for their patients. Pharmacist leaders emphasized the positive impact of COPD CARE delivery on VAMC metrics, while clinicians described how the program helped improve health outcomes for their patients. These tangible improvements, especially in care transitions post-discharge, incentivized both clinical support and resource allocation. This finding is consistent with the literature, showing that strong organizational commitment and value congruence lead to enhanced practice adoption and sustainment (16, 22). Implementation of the COPD CARE program at the medical centers included in this evaluation was supported by FTE funding. However, the future availability of these resources is uncertain; reduced funding may affect staff ability to sustain COPD CARE.

Clinical pharmacists were crucial to sustaining COPD CARE. Interprofessional team members (physicians, nurses, and senior leaders) highlighted how pharmacists addressed COPD workforce shortages, thereby promoting patient access to timely care. The expertise of pharmacists to optimize COPD management was also described as filling gaps in clinical care delivery. Prior studies have described pharmacist roles as completing tasks that are narrow in scope, such as providing inhaler technique education and adherence counseling to patients (23, 24). The success of the COPD CARE program argues for increasing the use of clinical pharmacists in more comprehensive roles.

### 4.1 Limitations and future directions

We view the inclusion of participants with direct involvement in the COPD CARE program as an evaluation strength that facilitates the identification of sustainment factors. At the same time, participants’ engagement with the program may have promoted social desirability bias when discussing their experiences with the interviewers. The inclusion of a broad participant group with unique roles and varying degrees of involvement in program delivery was intended to minimize this risk. The evaluation team recognized the potential for confirmation bias to influence the assessment process. To minimize the impact of confirmation bias, the evaluation team interviewers were intentionally selected because they were not VA employees and had limited prior engagement with VA assessments.

While VA has uniquely positioned pharmacists within primary care teams as prescribers, the team based primary care delivery model that exists within VA has not been widely scaled across non-VA healthcare settings. As a result, opportunities exist to better understand sustainment factors of team-based care models widely scaled outside of the VA. In addition, this evaluation was conducted in rural healthcare settings where known limitations to healthcare resources exist. Opportunities exist to better understand and compare sustainment factors for team-based COPD delivery models at medical centers in both urban and rural settings.

This evaluation includes stakeholder perspectives at 18 months following program implementation. Future research should examine factors that support program delivery sustainment beyond 18 months. While long-term follow-up is needed to assess long-term sustainment, the identified sustainment facilitators suggest the potential for long-term viability of COPD CARE. The program's adaptability allows for ongoing opportunities to optimize workflow and effectively utilize available resources, while institutionalization of COPD CARE into care delivery processes helps overcome high staff turnover, a barrier that hinders continuity of care and threatens program adherence over time. Opportunities exist to use the team-based approach to COPD management described in this paper and elsewhere (6–11) as a blueprint for expanding other COPD programs across the rural US healthcare landscape.

## 5 Conclusion

This evaluation explored facilitators of COPD CARE sustainment at two high-performing rural VA medical centers. Five key facilitators were identified: value alignment, leadership support, service institutionalization, service adaptation, and interprofessional collaboration. This evaluation demonstrated the impact of clinical pharmacists serving as prescribers in improving COPD management in rural settings.

## Data Availability

The raw data supporting the conclusions of this article will be made available by the authors, without undue reservation.

## References

[1] Ai-Ping C Lee KH Lim TK. In-hospital and 5-year mortality of patients treated in the ICU for acute exacerbation of COPD: a retrospective study. Chest. (2005) 128(2):518–24. 10.1378/chest.128.2.518 16100133

[2] Croft JB Wheaton AG Liu Y Xu F Lu H Matthews KA Urban-rural county and state differences in chronic obstructive pulmonary disease — United States, 2015. MMWR Morb Mortal Wkly Rep. (2023) 67(7):205–11. 10.15585/mmwr.mm6707a1 PMC5858043 29470455

[3] Linderman DJ Koff PB Min SJ Freitag TJ James SS Gunnison L Rural/urban health and treatment disparities in advanced chronic obstructive pulmonary disease. Chest. (2009) 136(4):90S. 10.1378/chest.136.4_MeetingAbstracts.90S-a

[4] Moore P Atkins GT Cramb S Croft JB Davis L Dolor RJ COPD And rural health: a dialogue on the national action plan. J Rural Health. (2019) 35(4):424. 10.1111/jrh.12346 30677167 PMC6790602

[5] National Heart, Lung, and Blood Institute. Making Strides to Address COPD in Rural Communities. Bethesda, MD: NIH (2023). Available online at: https://www.nhlbi.nih.gov/news/2023/making-strides-address-copd-rural-communities (Accessed July 5, 2025).

[6] Portillo EC Wilcox A Seckel E Margolis A Montgomery J Balasubramanian P Reducing COPD readmission rates: using a COPD care service during care transitions. Fed Pract. (2018) 35(11):30. 30766329 PMC6366592

[7] Portillo E Lehmann M Hagen T Maurer M Kettner J Bhardwaj S Evaluation of an implementation package to deliver the COPD CARE service. BMJ Open Qual. (2023) 12(1):2074. 10.1136/bmjoq-2022-002074 PMC9972453 36849192

[8] Portillo EC Lehmann MR Hagen TL Costner MG Kettner JT Bhardwaj SD Integration of the patient-centered medical home to deliver a care bundle for chronic obstructive pulmonary disease management. J Am Pharm Assoc (2003). (2023) 63(1):212–9. 10.1016/j.japh.2022.10.003

[9] Portillo EC Gruber S Lehmann M Kies K Margolis A Kreyer K Application of the replicating effective programs framework to design a COPD training program. J Am Pharm Assoc (2003). (2021) 61(2):e129–35. 10.1016/j.japh.2020.10.023 33309066

[10] Portillo EC Maurer MA Kettner JT Bhardwaj SD Zhang Z Sedgwick C Applying RE-AIM to examine the impact of an implementation facilitation package to scale up a program for veterans with chronic obstructive pulmonary disease. Implement Sci Commun. (2023) 4(1):1–10. 10.1186/s43058-023-00520-5 37990241 PMC10664371

[11] Erdelt D Portillo E Ourth H Hey JV Donovan L McFarland MS 1272 Evaluating the pace of implementation and sustainment within an interprofessional COPD service. J Am Pharm Assoc. (2025) 65(5):102735. 10.1016/j.japh.2025.102735

[12] Proctor E Luke D Calhoun A McMillen C Brownson R McCrary S Sustainability of evidence-based healthcare: research agenda, methodological advances, and infrastructure support. Implement Sci. (2015) 10(1):88. 10.1186/s13012-015-0274-5 26062907 PMC4494699

[13] Wiltsey Stirman S Kimberly J Cook N Calloway A Castro F Charns M. The sustainability of new programs and innovations: a review of the empirical literature and recommendations for future research. Implement Sci. (2012) 7(1):1–19. 10.1186/1748-5908-7-17 PMC3317864 22417162

[14] Feldstein AC Glasgow RE. A practical, robust implementation and sustainability model (PRISM) for integrating research findings into practice. Joint Commission J Qual Patient Safety. (2008) 34(4):228–43. 10.1016/S1553-7250(08)34030-6 18468362

[15] Vauterin D Van Vaerenbergh F Grymonprez M Vanoverschelde A Lahousse L. Medication adherence to inhalation therapy and the risk of COPD exacerbations: a systematic review with meta-analysis. BMJ Open Respir Res. (2024) 11(1):e001964. 10.1136/bmjresp-2023-001964 39304207 PMC11418573

[16] Hamilton AB Brunner J Cain C Chuang E Luger TM Canelo I Engaging multilevel stakeholders in an implementation trial of evidence-based quality improvement in VA women’s health primary care. Transl Behav Med. (2017) 7(3):478. 10.1007/s13142-017-0501-5 28585163 PMC5645285

[17] Williams NJ Wolk CB Becker-Haimes EM Beidas RS. Testing a theory of strategic implementation leadership, implementation climate, and clinicians’ use of evidence-based practice: a 5-year panel analysis. Implement Sci. (2020) 15(1):1–15. 10.1186/s13012-020-0970-7 32033575 PMC7006179

[18] Cowie J Nicoll A Dimova ED Campbell P Duncan EA. The barriers and facilitators influencing the sustainability of hospital-based interventions: a systematic review. BMC Health Serv Res. (2020) 20(1):1–27. 10.1186/s12913-020-05434-9 PMC7321537 32594912

[19] Miller CJ Sullivan JL Connolly SL Richardson EJ Stolzmann KL Brown M Adaptation for sustainability in an implementation trial of team-based collaborative care. Implement Res Pract. (2024) 5:26334895231226197. 10.1177/26334895231226197 38322803 PMC10807389

[20] von Thiele Schwarz U Giannotta F Neher M Zetterlund J Hasson H. Professionals’ management of the fidelity–adaptation dilemma in the use of evidence-based interventions—an intervention study. Implement Sci Commun. (2021) 2(1):1–9. 10.1186/s43058-021-00131-y 33726864 PMC7962232

[21] Nathan N Shelton RC Laur C V Hailemariam M Hall A. Editorial: sustaining the implementation of evidence-based interventions in clinical and community settings. Front Health Serv. (2023) 3:1176023. 10.3389/frhs.2023.1176023 37033900 PMC10080155

[22] Tiu VM Picardo RL. Beliefs, organizational culture and readiness on implementation of evidence-based practice among nurses in selected district hospitals. Int J Res Sci Innov. (2025) XII(III):273–93. 10.51244/IJRSI.2025.12030018

[23] Mayzel B Muench S Lauster C. Impact of pharmacist education on inhaler technique and adherence in an outpatient clinic. Hosp Pharm. (2021) 57(3):402. 10.1177/00185787211046863 35615485 PMC9125121

[24] Kebede AT Trapnes E Lea M Abrahamsen B Mathiesen L. Effect of pharmacist-led inhaler technique assessment service on readmissions in hospitalized COPD patients: a randomized, controlled pilot study. BMC Pulm Med. (2022) 22(1):210. 10.1186/s12890-022-02004-z 35624509 PMC9145163

